# Methodological influences on circulating cell-free-mitochondrial and nuclear DNA concentrations in response to chronic stress

**DOI:** 10.1007/s11033-025-10369-7

**Published:** 2025-03-13

**Authors:** Carina Daubermann, Benedict Herhaus, Elmo W. I. Neuberger, Perikles Simon, Katja Petrowski

**Affiliations:** 1 Department of Sports Medicine, Rehabilitation and Disease Prevention, Johannes Gutenberg University Mainz, Albert-Schweitzer Strasse 22, 55128 Mainz, Germany; 2 Department of Medical Psychology and Medical Sociology, University Medical Centre of the Johannes Gutenberg-University, Duesbergweg 6, 55128 Mainz, Germany

**Keywords:** Circulating Cell Free mitochondrial DNA, Circulating cell free nuclear DNA, Chronic Stress, Biomarker, Assay development

## Abstract

**Background:**

Mitochondria are versatile eukaryotic organelles that play a crucial role in the body’s stress response. Prolonged stress exposure can cause structural and functional alterations, leading to mitochondrial DNA (mtDNA) damage and subsequent release of mtDNA into the circulation. Cell-free circulating mtDNA (ccf-mtDNA) is a potential biomarker indicating cellular damage and stress. In this study we investigated the applicability of ccf-mtDNA and cf-nDNA as biomarkers of chronic stress in healthy subjects.

**Methods and results:**

We developed a quantitative polymerase chain reaction (qPCR) assay to directly measure ccf-mtDNA in human blood plasma samples, addressing numerous challenges specifically related to ccf-mtDNA quantification. We validated our 68 bp target assay based on the FDA, International Organization for Standardization (ISO) and Clinical & Laboratory Standards Institute (CLSI) guidelines for assay development, including parameters such as limit of blank (LOB), limit of detection (LOD) and limit of quantification (LOQ). Furthermore, we implemented incurred samples analysis and inter-plate samples to ensure reliability and reproducibility of the assay. In addition, we evaluated the effects of centrifugation forces on ccf-mtDNA and cf-nDNA concentrations in native plasma samples and showed that mainly ccf-mtDNA is strongly affected by centrifugation forces. We found a significant negative correlation between ccf-mtDNA levels and chronic stress. In contrast, cf-nDNA levels were not affected in response to chronic stress.

**Conclusion:**

ccf-mtDNA can directly and reliably quantified in unpurified plasma samples. However, the ccf-mtDNA levels in plasma samples of healthy subjects are close the LOQ, showing that the assay is not yet suitable for all conditions.

**Supplementary Information:**

The online version contains supplementary material available at 10.1007/s11033-025-10369-7.

## Introduction

In 2023 the American Psychological Association [1] found an increase in mental and physical health issues due to long-term stressors. Chronic diseases increased from 48% in 2019 to 58% in 2023, while mental health diagnoses increased from 31 to 45%, reaching 50% among young adults (18–34) [1]. Therefore, quantifiable biological indicators that offer critical insights into the effect of acute and chronic stress on the body would be of high value.

The stress response is an evolutionary adaptive mechanism designed to enable individuals to cope with acute threats, often referred to as the “fight-or-flight” response. Stress activates the hypothalamic–pituitary–adrenal (HPA) axis and the sympathetic nervous system, releasing hormones such as cortisol and adrenaline to increase heart rate, focus attention and mobilize energy for rapid action. However, when stress becomes chronic—whether through ongoing or repeated exposure to stressors or inadequate/insufficient coping strategies, or a combination of the two—the body remains in a state of heightened arousal, which can have a range of negative physical and mental health consequences [2]. Prolonged activation of the HPA axis results in allostatic load, resulting in immune dysregulation, inflammation, oxidative stress, and impacts on cardiovascular and metabolic health [3]. It can therefore be assumed that stress plays an important role in the dysregulation of immune and metabolic functions, contributing to a range of chronic health conditions. One key aspect of this process is mitochondrial function, which is tightly linked to cellular stress and immune responses [4, 5].

As the energy supply centers of the cell, mitochondria are essential for maintaining bioenergetics, but they are also highly responsive to stress signals to adapt to fluctuating energy demands during stressful situations. Regulating their functions to cope with ongoing cellular stress is a phenomenon known as mitochondrial allostatic load [6]. One consequence of mitochondrial stress is the release of mitochondrial DNA (mtDNA) into the circulation, referred to as circulating cell-free mtDNA (ccf-mtDNA). This release can occur by two different mechanisms: passive release resulting from dysfunctional mitochondria, cell death or cell damage and active secretion as part of regulated signaling processes. The main sources for ccf-mtDNA by passive mechanisms are necrosis and apoptosis [7] and research has shown that elevated ccf-mtDNA levels can be found in conditions such as cancer [7], sepsis [8] and trauma, where tissue damage can contribute to the passive release. In contrast, active secretion can enable intercellular and mitochondrial communication [9] through the secretion of either whole naked mitochondria or mitochondrial components encapsulated within extracellular vesicles [10]. This process typically occurs when mitochondrial function is compromised and needs to be repaired to maintain cellular bioenergetics and overall cellular health [10]. In some cases, mtDNA can also escape into the circulation as a result of immune responses. For example, immune cells such as neutrophils, release mtDNA when they form neutrophil extracellular traps [11] in response to pathogens such as bacteria or fungi.

Since mitochondria originated from alpha-proteobacteria approximately two billion years ago [12], their mtDNA is closely related to bacterial DNA and can act as an activator of the innate immune system and a potent inflammatory trigger, by binding receptors such as Toll-like receptor 9, thereby increasing pro-inflammatory cytokine production [13]. However this process must be tightly regulated to mitigate the effect of excessive immune activation. It can therefore be assumed that different pathologies may result in different mtDNA release mechanisms, influencing disease progression and immune responses.

Unlike circulating cell- free nuclear DNA (cf-nDNA), which is associated with a variety of pathological conditions, including physical and psychosocial stress [14, 15], ccf-mtDNA specifically reflects mitochondrial-related impairments [16]. As a result to cumulative stress, recent research indicates ccf-mtDNA as a potential biomarker in assessing acute as well as chronic psychological or psychosocial stress [17, 18].

However, challenges exist in detection methods due to the presence of nuclear-embedded mtDNA sequences (NUMTs) [19], variations in fragment sizes [20], and no defined guidelines regarding preanalytical considerations. Based on our previous work [21], we developed an assay for the direct quantification of ccf-mtDNA concentrations in human blood plasma samples without DNA extraction. For our quantitative polymerase chain reaction (qPCR)-based approach, we have implemented the recommendations for the development of qPCR and digital polymerase chain reaction (dPCR) assays in accordance with the bioanalytical method validation guidelines of the Food and Drug Administration (FDA) [22], Clinical & Laboratory Standards Institute (CLSI) [23] and International Organization for Standardisation (ISO) guidelines [24] concerning specificity, reproducibility, reliability, limit of quantification (LOQ), limit of detection (LOD), limit of blank (LOB) and coefficient of variation (CV). Furthermore, we addressed the previously described challenges associated with ccf-mtDNA quantification, including targeting only mtDNA without amplification of NUMTs and detection of small fragments < 70 bp. This offers the potential to monitor mitochondrial functionality in pathological conditions as mentioned above and help manage disease severity and outcome.

In this study, we aimed to measure ccf-mtDNA and cf-nDNA levels in blood plasma samples from 22 healthy volunteers in respect to their chronic stress load. We evaluated the efficiency of a silica column-based isolation kit and compared our direct quantification approach with traditional methods using extracted samples. Furthermore, we compared three different centrifugation protocols to address preanalytical considerations related to ccf-mtDNA, which is crucial for accuracy and reliability of the measurements. Our findings provide insights into optimized methodologies for ccf-mtDNA quantification and highlight the importance of standardized protocols in biomarker research and assay development.

## Material and methods

### Study participants

Twenty-two healthy female (n = 19) and male (n = 3) participants were recruited through electronic announcements at the Johannes Gutenberg University Mainz. Eligibility criteria were evaluated via telephone interviews using the Structured Clinical Interview (SCID) [25] based on the Diagnostic and Statistical Manual of Mental Disorders, Fourth Edition (DSM-IV) [26]. Exclusion criteria included the presence of acute or chronic medical conditions, mental health disorders, medication or substance use, significant stressful life events within the past six months and smoking more than ten cigarettes daily. The average age of participants was 37.55 ± 13.80 years, with a mean body mass index (BMI) of 24.56 ± 3.14 kg/m^2^. The study protocol was approved by the local Ethics Committee of the Landesärztekammer Rheinland-Pfalz, Germany (No#2019–14188).

### Study protocol

Blood samples were collected following a 30-min stationary period between 2:00 p.m. and 5:00 p.m. During this period, participants had the option to read magazines. Prior to blood collection, participants completed the Trier Inventory for Chronic Stress questionnaire [27]. The participants were asked to refrain from eating, drinking, and smoking for at least two hours before blood collection.

### Evaluation of chronic psychological stress

The German short version of the Trier Inventory for Chronic Stress (TICS-9) [27] was used to measure the subjective perception of chronic stress in the previous three months. Nine items have to be answered on a five-point rating scale ranging from ‘never’ (0) to ‘very often’ (4) [27]. A validation study with 2.473 women and men showed good reliability with an internal consistency value (Cronbach’s Alpha-coefficient) of α = 0.88 [27].

### Blood sample collection and preparation

Venous blood samples were collected in 9 mL tripotassium ethylenediaminetetraacetic acid (K3 EDTA)-monovettes (Sarstedt, Nümbrecht, Germany). Immediately after collection, the samples were centrifuged at room temperature. For comparison studies of centrifugation protocols, the blood samples underwent a three-step centrifugation process: first at 600 × *g* for 15 min, aliquoted and centrifuged again at 2500 × *g* for 15 min, aliquoted once more, and finally centrifuged at 16,000 × *g* for 15 min. All plasma aliquots were stored at – 20 °C.

### Assay validation material for ND1 assay

Linearity and accuracy of the assay was tested on a custom-made 102 bp fragment of the human mitochondrial *ND1*-gene (Table 1) (NCBI Reference Sequence: NC_012920.1). The fragment was synthesized by Eurofins MWG Operon (Eurofins MWG Operon, Ebersberg, Germany). The concentration was determined with a NanoDrop 3300 fluorospectrometer (Thermo Fisher Scientific, Inc., Waltham, MA) using Quant-iT PicoGreen dye (Thermo Fisher Scientific). Two calibration samples were prepared by spiking mouse plasma with sonicated DNA at known concentrations, resulting in a final dilution of 1:10. The DNA was isolated from 30 mL of pooled whole blood from four healthy donors using the Qiagen Puregene Blood Core Kit B (Qiagen, Hilden, Germany). DNA was sonicated with the Covaris S220 system (Covaris) using a microTUBE AFA Fiber Pre-Slit Snap-Cap 6 × 16mm tube according to the manufactures instructions for DNA shearing with microTUBES for 400 bp base pair peak. The reference samples were aliquoted in 20 µL and stored at − 20 °C.

**Table 1:**
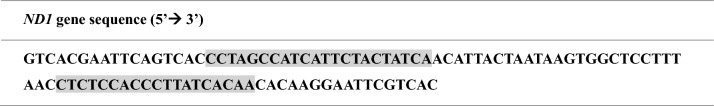
Sequence of the custom made human *ND1* gene containing an *Eco*RI restriction site. Reverse and forward primer binding sites are highlighted in grey

### Primer design

Due to the highly hypervariable nature of mtDNA in certain regions [28], primers were designed according to the following criteria: (1) targeting conserved rather than hypervariable regions to ensure specificity (2) minimizing the risk of NUMTs co-amplification (3) keeping the target size below 70 bp to optimize efficient amplification of small fragments.

We performed a local alignment analysis with the NCBI primer BLAST [29] to show the specificity of the primer set. Furthermore, we checked for secondary structures including heterodimers and hairpins using the OligoAnalyzer™ Tool [30].

### Comparison of direct quantification and isolation

Ccf-mtDNA was isolated from 200 µL of the three-times centrifuged plasma samples using the QIAamp DNA Blood Mini Kit (Qiagen) according to the manufacturer’s instructions for body fluids. DNA was then eluted in a final volume of 50 µL H_2_O. The extracts were stored at – 20 °C immediately after isolation.

### Sample preparation and quantification of ccf-mtDNA and cf-nDNA in plasma

Quantification of cf-nDNA was based on the amplification of the repetitive human long interspersed nuclear element 1 (LINE1) (GRCh38/hg38_ chr4:68,085,016–68,085,410 / size = 395 bp / strand = +) targeting a 90 bp fragment of the gene (5′-TGCCGCAATAAACATACGTG-3′ and 5′-GACCCAGCCATCCCATTAC-3′). Detailed protocol, assay precision and specifications can be found in Neuberger et al*.* [21]. In brief, plasma samples were diluted 1:15 in UltraPure DNase/RNase-free H_2_O (Invitrogen, Waltham, MA), each sample was measured in a final volume of 5 µL in technical replicates of three. qPCR mix consisted of 1 µL 1:15 diluted plasma sample, 0.1 µL primer mix (140 nM final concentration of each primer) and 3.9 µL of master mix. Final master mix concentrations were 1.2 × MegaFi Pro Reaction Buffer (BioCat GmbH, Heidelberg, Germany) 0.3 mM of each dNTP (BioCat GmbH), 0.15 × SYBR Green (Sigma-Aldrich, Taufenkirchen, Germany) and 0.05 U MegaFi Pro Fidelity DNA Polymerase (BioCat GmbH). The pipetting was performed by a pipetting robot (Assist Plus, Integra).

For the absolute quantification of ccf-mtDNA, plasma samples were diluted 1:10 in UltraPure DNase/RNase-free H_2_O (Invitrogen) and measured using direct qPCR. Extracts remained undiluted. 3 µL of diluted plasma sample or extract was mixed with 11.7 µL of Master Mix and 0.3 µL of Primer Mix and measured in triplicates in a final volume of 5 µL. Final PCR Mix concentrations were 1.2 × MegaFi Pro Reaction Buffer (BioCat GmbH) 0.3 mM of each dNTP (BioCat GmbH), 0.2 × SYBR Green (Sigma-Aldrich), 0.05 U MegaFi Pro Fidelity DNA Polymerase (BioCat GmbH) and 300 nM of forward (for: 5′-CCTAGCCATCATTCTACTATCA-3′) and reverse (rev: 5′-TTGTGATAAGGGTGGAGAG-3-′) primers.

Amplification was performed on a Bio-Rad CFX384 system thermocycler (Bio-Rad, Hercules, CA, USA) with the following conditions: 95 °C for 5 min, followed by 35 cycles of 95 °C for 10 s and 60 °C for 15 s including a plate reading step. A melt curve analysis from 60–90 °C with an increase of 0.5 °C every 10 s was performed in each run. If the quantification cycle (Cq) of the triplicates showed higher standard deviation (SD) than > 0.4, the plasma samples were re-diluted and re-analyzed.

### Determination of assay performance and reproducibility of ND1 assay

For the determination of LOQ and LOD three standard curves were generated in water and mouse plasma. Mouse plasma is a suitable background matrix mimicking the inhibition of real native human plasma without containing the human specific mitochondrial *ND1* gene sequence. The mouse plasma was spiked with a custom-made fragment of the *ND1* gene covering a dynamic range of 1 × 10^6^–25 copies/PCR. Final dilution of the spike in samples were 1:10 in water. The standard curve measurements were carried out in seven replicates on three different days. As non-template control (NTC) H_2_O and 1:10 diluted mouse plasma was included in each run. Additionally, the two calibrator samples were included in each run on each plate. According to the CLSI- guideline EP17-A [23], LOB and LOD were defined as followed:

LOB = mean_blank_ + 1.645(SD_blank_).

LOD = LOB + 1.645 x (SD _low copy number sample)_ [23].

Reproducibility of the assay was evaluated based on the mean Cq values of the LOQ curves, with the CV calculated using the following equation:

$$ CV = \left( {\frac{SD}{{Mean}}} \right) \times 100 $$

### Incurred sample reanalysis

The reliability of the assay was determined by re-analyzing a subset of diluted plasma samples in two different runs. The percentage difference between the first and repeated measurement was calculated using the following equation:

$$ \frac{(repeated\,measurement - first\,measurement)}{{mean}} \times 100 $$

According to the FDA Bioanalytical Method Validation Guidance for Industry [22].

### Normalization strategy—Inter-run calibration

The two calibrator samples were used to normalize the data and correct for inter-run differences, minimize the impact of technical variations and reduce background fluorescence noise. The reference samples were measured in seven replicates in three independent runs including the LOQ curve. The mean Cq values of all measurements were used for threshold adaption. Reference samples were aliquoted in 20 µL and stored at – 20 °C. Freeze–thaw cycles were limited to three.

### Calculation of ccf-mtDNA and cfnDNA

To calculate the ccf-mtDNA concentrations in copies/mL from the measured Cq values the following equation was used:

$$ \frac{copies}{{mL}} = 10^{{\frac{(Cq - yIntercept)}{{slope}}}} /5\mu L/0.02\,or\,0.2 \times 1000 $$

For plasma samples and extracts, the equation includes a total dilution factor of 0.02 and 0.2, respectively. This factor accounts for the initial 1:10 dilution of the plasma samples and the additional dilution in the qPCR reaction (3 µL sample in a 15 µL reaction volume). Division by 5 equals the copies/µL. Multiplication by 1000 gives the number of copies/mL. An elution factor of four was estimated for the final concentrations of the extracted samples (200 µL native plasma sample eluted in 50 µL H_2_O). cf-nDNA concentrations were calculated according to the equation described in Neuberger et al. [21].

### Data analysis

The qPCR analysis was performed with the Bio-Rad CFX Maestro software version 2.3 (Bio-Rad, Hercules, CA, USA) and Microsoft® Excel, 2016. For statistical analyses and graphical illustration RStudio (v4.3.3) with ggplot package [31] was used. Imprecision profile was generated using the R VFP package (v1.4.1). The data sets were transformed using log10 and tested for normal distribution using Shapiro–Wilk test. Pearson’s and Spearman’s correlation test were used for normal distributed and not normally distributed data, respectively. Wilcoxon rang-sum test was used as nonparametric statistical test for non-normal distributed data. *P*-values < 0.05 were considered significant.

## Results

### mtDNA assay performance

**Fig. 1:**
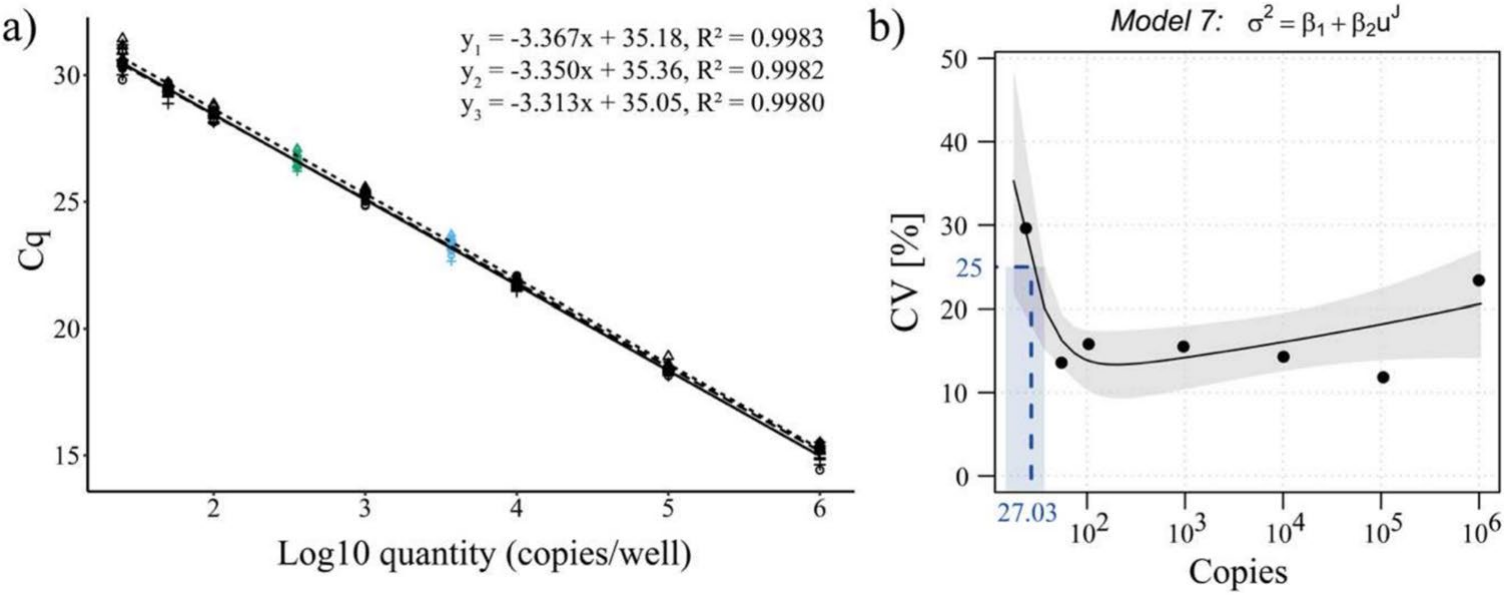
LOQ curves and imprecision profile of *ND1* assay. Three standard curves were measured in septettes for each concentration, with green and blue dots representing the two calibrator samples and black dots representing the standards **a** Imprecision profile was generated using R VFP package, based on the formula σ^2^ = 35.68 + 0.0095 × U^2.108^ **b** Figures were produced using the R ggplot2 package

**Fig. 2:**
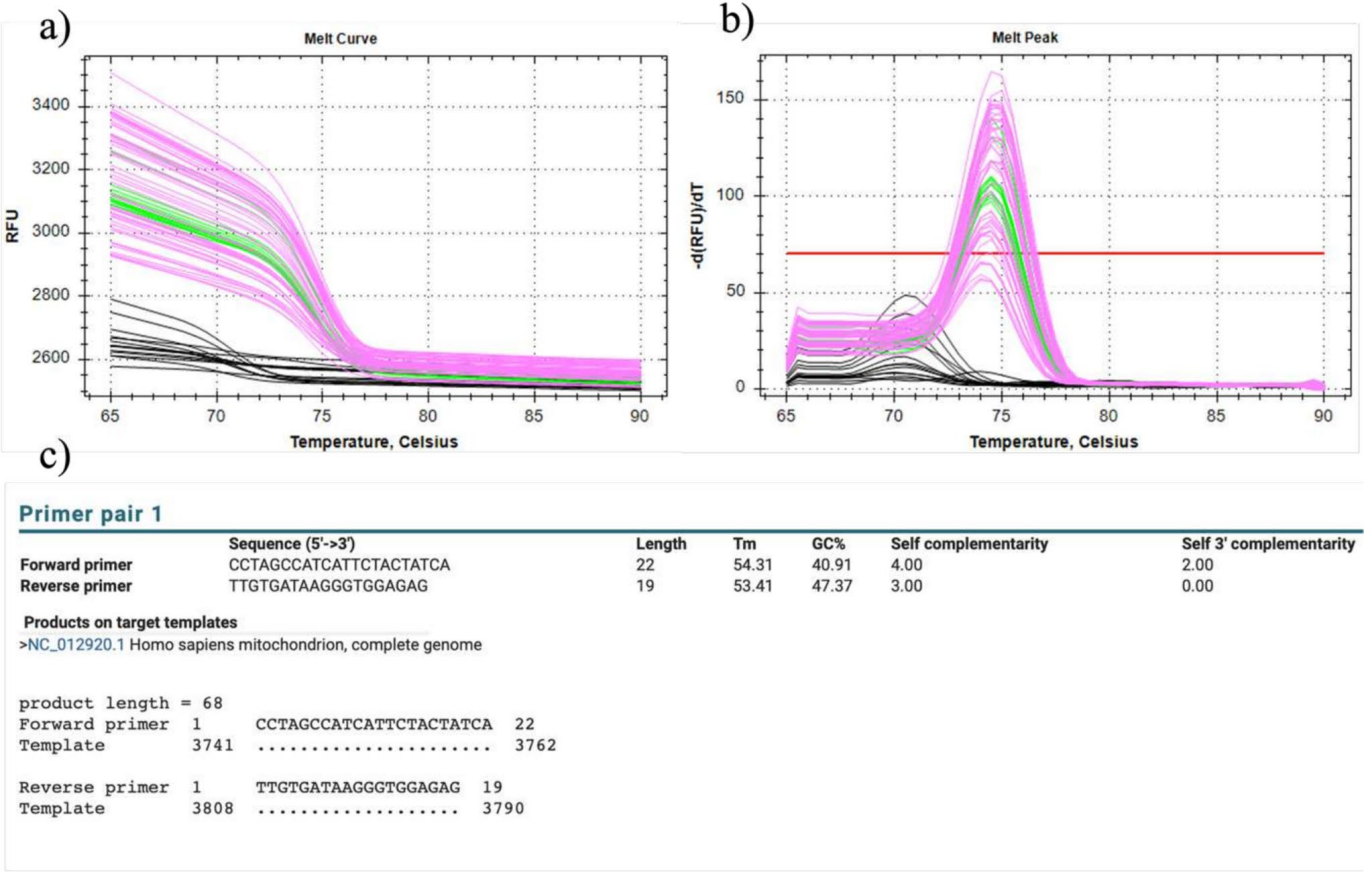
Specification of the *ND1* assay. Melt curve and melt curve peak of the *ND1* target fragment (**a**, **b**). Pink and green lines indicate samples used to generate the standard curve and calibrators, respectively (**a**, **b**). Black lines indicating primer dimers in the non-template controls (**a**, **b**). Extract of the BLAST results of the primers used for the *ND1* assay, showing specificity and accuracy of the primers with no off-target amplification (**c**). (Color figure online)

To evaluate the performance of the newly designed mtDNA assay three independent standard curves were generated using the custom-made fragment of the *ND1* gene. The values obtained from these measurements are provided in the Supplementary Information Table S3. The data was positively tested for normal distribution using Shapiro–Wilk test. The three independent standard curves have similar y-intercept and slope as illustrated in Fig. 1a. Efficiencies are ranging between 98.15% and 100.38%, while linearity shows R^2^ > 0.99 (Supplementary Information Fig. S1, Tab. S4). LOB and LOD were calculated according to the CLSI guideline, yielding a LOB of 4.4 copies and a LOD of 15.41 copies (Supplementary Information Tab. S6). There are no defined guidelines for LOQ determination, but following the recommendations for qualitative real-time PCR methods [32], a CV of ≤ 25% was set as the threshold for LOQ. Values are included in the Supplementary Information Tab. S5. The imprecision profile of the assay showed a CV below 25%, meeting the predefined threshold for precision. The LOQ of 27.03 copies/PCR was then determined based on the imprecision profile (Fig. 1b). All replicates of the low copy number sample were successfully detected within the assay range (10^6^ – 27.03 copies/PCR), remaining above the LOD. Specificity of the assay was confirmed by melt curve analysis and local alignment analysis with BLAST (Fig. 2).

### Incurred sample realanysis

**Fig. 3:**
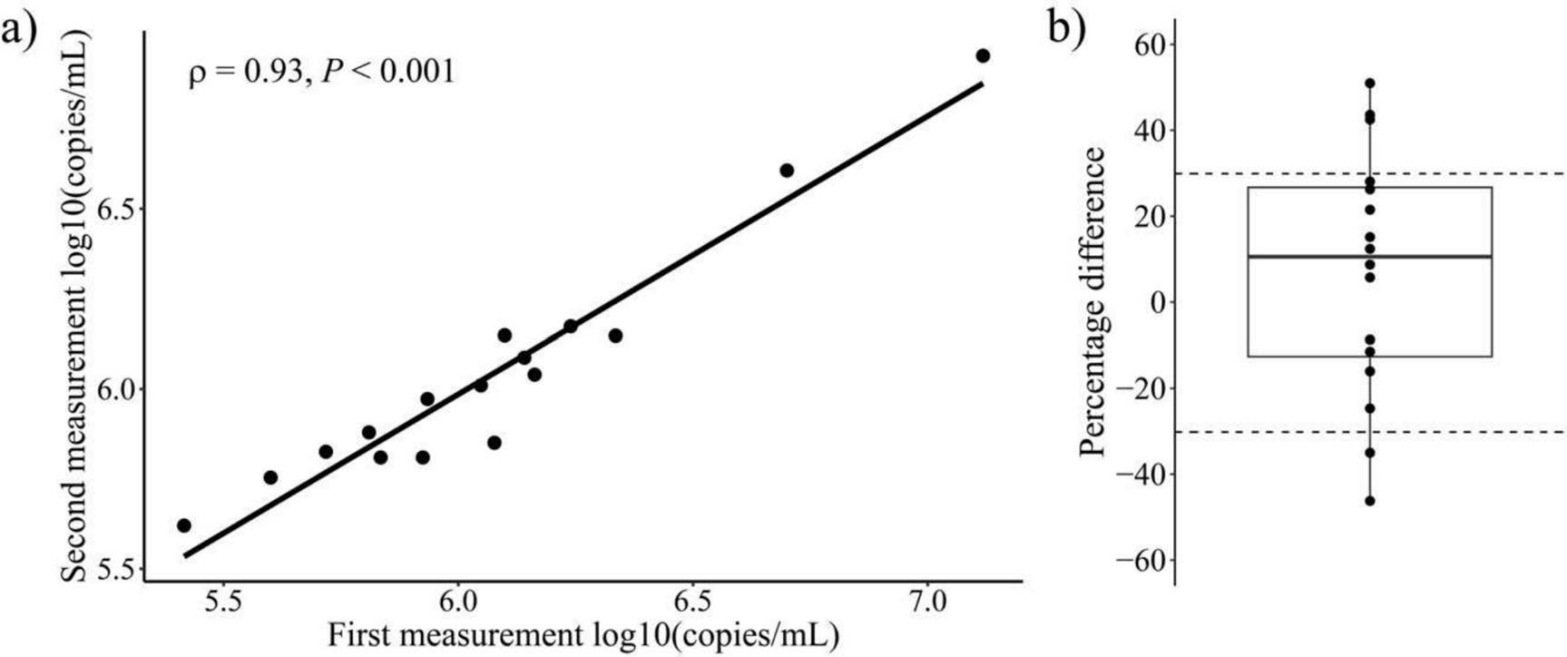
Precision of *ND1* assay. Correlation between initial and repeated measurements of diluted plasma samples **a** Percentage difference between initial and repeated measurements **b** Figures were produced using the R ggplot2 package

To evaluate the accuracy and reliability of the mtDNA assay, approximately 29% of the diluted plasma samples were re-analyzed in two different runs (Supplementary Information Tab. S2).The ccf-mtDNA concentrations showed a strong correlation between the initial and repeated measurements (r = 0.93, *p* < 0.001) (Fig. 3). According to the FDA Guidelines for Bioanalytical Method Validation [22], two-thirds of the reanalyzed samples should exhibit a percentage difference of less than 30%. In this subset, 5 samples (31.25%) exceeded the 30% threshold, while the remaining samples were within the acceptable range (Fig. 3).

### Kit isolation reduces ccf-mtDNA concentrations

**Fig. 4:**
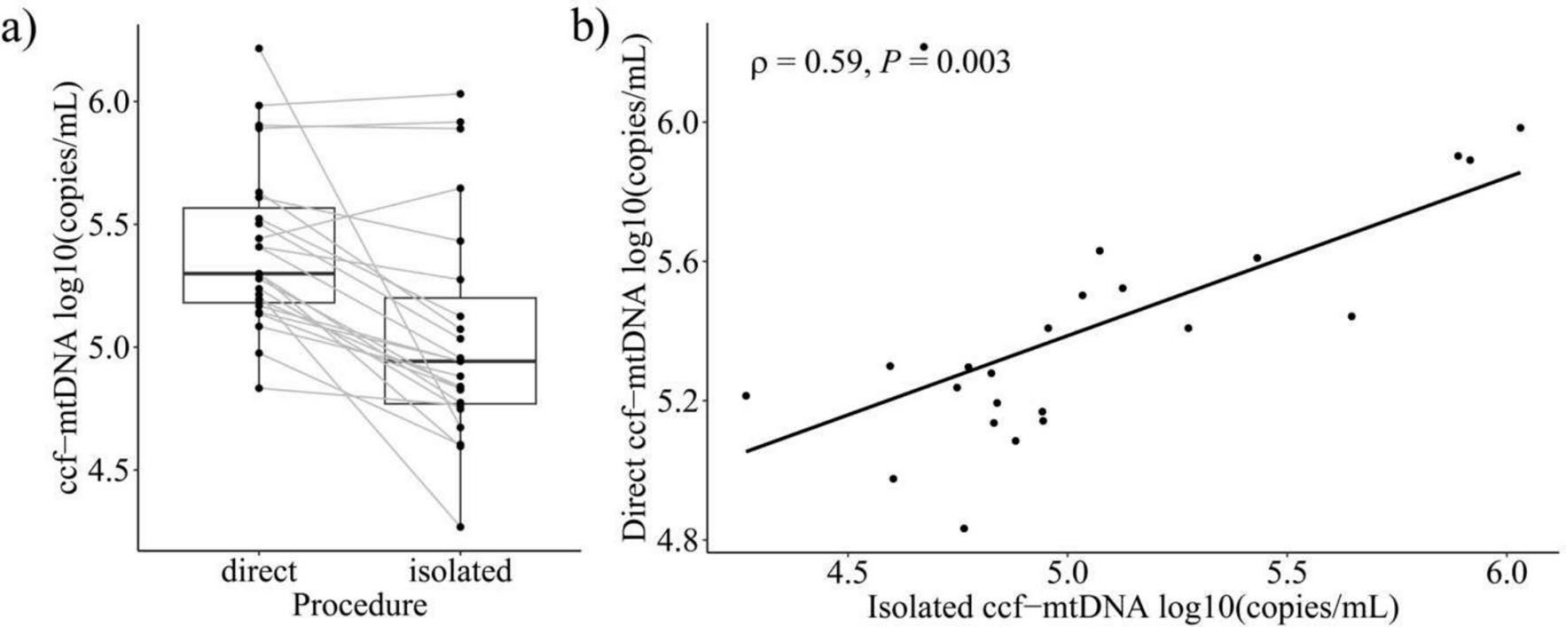
Effects of Isolation. Comparison of direct quantification and silica column based isolated samples **a** Correlation between direct and isolated samples **b** Figures were produced using the R ggplot2 package

To compare our direct quantification approach with a kit isolation method, ccf-mtDNA was directly quantified in a subset of diluted plasma samples and compared with the corresponding isolated samples (Supplementary Information Table S1, S2). The comparison of direct quantification with isolated samples revealed a notable loss of approximately 43.3% (± 38.0) during isolation using the QIAamp DNA Blood Mini Kit (Fig. 4a). Direct and isolated measurements correlated positively, with a Spearman’s correlation coefficient of 0.59 (Fig. 4b).

### Centrifugation forces affect ccf-mtDNA levels

**Fig. 5:**
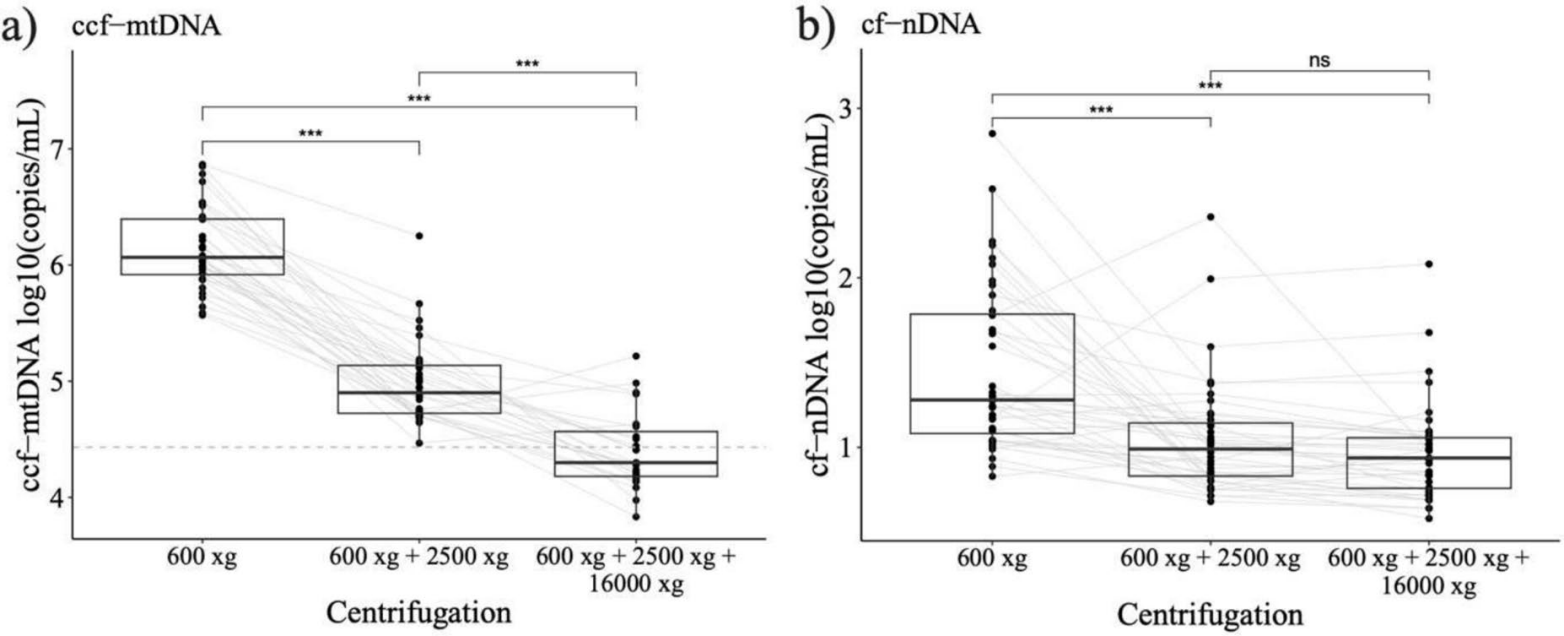
Effects of centrifugation forces. Effects of different centrifugation protocols on ccf-mtDNA concentrations in unpurified plasma, with the dotted line indicating the LOQ of the assay **a** Effects of different centrifugation protocols on cf-nDNA concentrations in unpurified plasma **b** Statistical significance levels are described as *p ≤ 0.05, **p ≤ 0.01 and ***p ≤ 0.001. Figures were produced using the R ggplot2 package

We analyzed the effect of different centrifugation forces on ccf-mtDNA concentrations in diluted native plasma samples (Supplementary Information Table S2). Ccf-mtDNA concentrations in native plasma samples significantly decreased with increasing centrifugation force (Fig. 5a). All samples centrifuged at 600 × *g* and 2500 × *g* remained within the quantification range, while only 45% of those centrifuged at 16,000 × *g* were within the assay LOQ (Fig. 5a). In contrast, a significant difference in cf-nDNA levels were found between 600 × *g* and 2500 × *g* and 600 × *g* and 16,000 × *g*, respectively (Fig. 5b).

### Ccf-mtDNA decreases with chronic stress but not cf-nDNA

**Fig. 6:**
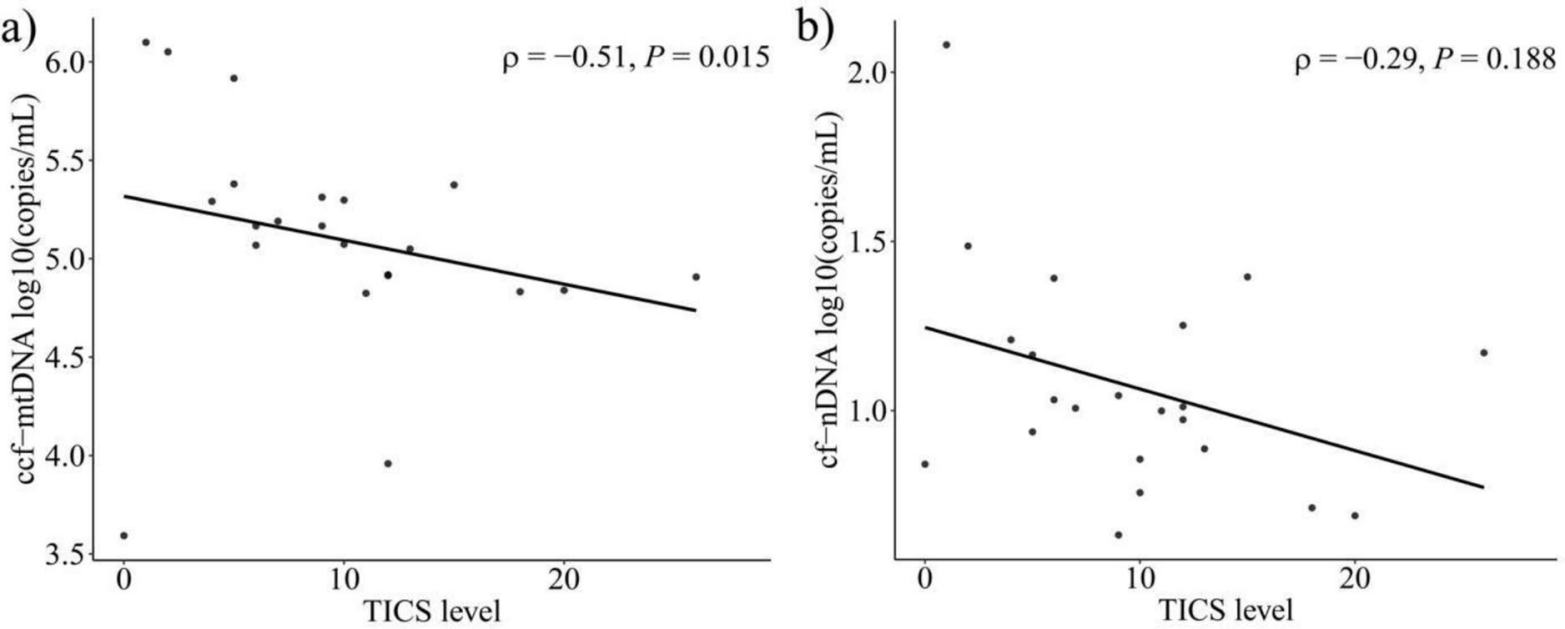
Effects of chronic stress on ccf-mtDNA (**a**) and cf-nDNA (**b**) in healthy subjects. Figures were produced using R ggplot2 package

As only 45% of the native plasma samples centrifuged at 16,000 × *g* remained above the LOQ threshold of the ccf-mtDNA assay, all samples were purified using the QIAamp DNA Blood Mini Kit (Supplementary Information Table S1). To further evaluate a potential relationship between stress and cell-free nucleic acids, we conducted the TICS-9 as a standardized questionnaire designed to assess the chronic stress load in our healthy cohort. Spearman’s rank correlation showed a significant negative association between the TICS level (Supplementary Information Table S7) and ccf-mtDNA levels in purified 16,000 × *g* centrifuged plasma samples, with a correlation coefficient of − 0.51 (Fig. 6a). However, no significant correlation was found between TICS levels and cf-nDNA in native unpurified 16,0000 × *g* plasma (Fig. 6b).

## Discussion

Over the past decade, cell-free nucleic acids have been recognized as a promising biomarker in liquid biopsy. Serval studies highlight its prognostic and diagnostic potential in pathological conditions including cancer [33], neurodegenerative diseases [34], mood disorders [35], and infectious conditions. Here we provide an assay for the absolute quantification of ccf-mtDNA directly in human blood plasma samples, with an LOQ of 27.03 copies (Fig. 1). Our study demonstrates that preanalytical aspects such as centrifugal forces and isolation strongly affects ccf-mtDNA measurement outcomes, but not cf-nDNA (Fig. 4, 5). Moreover, we used the new assay to quantify ccf-mtDNA in healthy subjects and found a significant reduction (*p* = 0.015) in ccf-mtDNA levels in response to chronic stress, while cf-nDNA concentrations remained unchanged (Fig. 6).

For the assay development we implemented the recommendations for developing qPCR and dPCR assays according to the ISO 20395:2019 [24], FDA [22] and CLSI EP17 -A guidelines [23]. The use of two inter-run calibration samples ensures the reliability of the assay and allows ccf-mtDNA concentrations to be compared across multiple runs on different plates. Reproducibility was verified with incurred sample reanalysis, demonstrating a strong correlation between the initial and repeated measurements, with concentrations remaining within the predefined range (Fig. 3). Our findings show that ccf-mtDNA can be directly quantified in human blood plasma samples without DNA extraction, offering a faster and more cost-effective alternative to bead- or column-based extraction methods for ccf-mtDNA quantification. Direct quantification not only reduces processing time, but also minimizes DNA loss, a common problem of isolation procedures, as we have demonstrated in this and previous studies [36] (Fig. 4). In particular, small DNA fragments, exhibit lower binding efficiency and may pass through columns during extraction or remain attached to the silica membrane, which can lead to biased results [37]. This is important because ccf-mtDNA predominantly consists of smaller fragments, ranging in size from 50 to 400 bp and peaking at approximately 80 bp [38]. In contrast, cf-nDNA exhibits larger fragments, with a peak around 166 bp [39], making ccf-mtDNA more sensitive to loss during conventional isolation procedures. To address this issue in our direct quantification approach, we targeted a 68-bp fragment of the human mitochondrial *ND1* gene, ensuring more accurate detection of smaller fragments.

When quantifying ccf-mtDNA the presence of NUMTs in the samples should be considered in primer and or probe design. NUMTs, which are incorporated into the nuclear genome, can be released as cf-nDNA into the circulation. Due to their high similarity to mtDNA, these sequences may inevitably be co-amplified during PCR if the primer or probe design lacks precision [19]. The specificity of our primers was confirmed by melt curve analysis and BLAST results (Fig. 2). The melt curve analysis demonstrated a distinct peak corresponding to the target, and the BLAST results showed a single, specific match in the mitochondrial genome, confirming the accuracy of our primer design (Fig. 2).

We have demonstrated that different centrifugation forces influence the abundance of ccf-mtDNA in human blood plasma samples (Fig. 5a), which aligns with previous studies [40, 41]. These differences might primarily occur due to particle-bound mtDNA, such as platelet-associated DNA. Platelets, the smallest human blood cells, play a central role in blood clotting and immune response. Although they lack nuclear DNA, they contain intact mitochondria and mtDNA. Numerous studies have consistently demonstrated that platelet counts in plasma samples are highly influenced by both centrifugation force and duration, with prolonged or high-speed centrifugation significantly reducing platelet levels and mtDNA in plasma samples [42–44]. In this study, plasma samples centrifuged at high speeds resulted in ccf-mtDNA concentrations close to or below the LOQ of the direct assay, making quantification at these speeds less reliable (Fig. 5a). However, it is important to note that higher centrifugation forces reduce platelet counts and therefore yield a “purer” form of free circulating mtDNA. In contrast, lower centrifugation forces (e.g., 2500 × *g*) allow for more consistent detection, as all samples remained within the assay’s LOQ for ccf-mtDNA. Despite these differences, a strong correlation was still observed between purified and unpurified ccf-mtDNA measurements at 16,000 × *g* (Fig. 4b), suggesting that while absolute values may differ, trends remain comparable. Additionally, using our direct quantification approach platelet and mitochondrial membranes will be disrupted during PCR denaturation in unpurified plasma samples, leading to the release of mtDNA and subsequently increased ccf-mtDNA measurements in the lower centrifuged plasma samples.

Therefore, we recommend a two-step centrifugation protocol prior to ccf-mtDNA analysis in plasma. The first step involves low-speed centrifugation (1000–1600 × *g*, 15 min) to remove blood cells and large particles, followed by high-speed centrifugation (e.g. 16,000 × *g*, 15 min). The first centrifugation separates the plasma, which should be transferred into a new tube, maintaining at least a one-centimeter gap from the buffy coat layer. The second centrifugation should be performed similarly, with both steps conducted at room temperature to avoid potential platelet activation and mtDNA release [45]. The blood should be centrifuged immediately after collection to avoid delays that could lead to cell degradation or inadvertent platelet activation. Roch et al. have shown that mtDNA levels can increase 67-fold when platelets are activated, highlighting the importance of careful handling during processing and the impact of platelets on ccf-mtDNA levels in plasma [46]. Although this procedure effectively removes cells, platelets, cellular debris and mitochondria, extracellular vesicles and exosomes, which usually pellet at ultra-high centrifugation speeds (≥ 100,000 × *g*) [47] will remain in the plasma, which should be considered for downstream analysis. However, this procedure enables to the capture the rather small proportion of ccf-mtDNA human plasma, ensuring that only the freely circulating DNA is isolated, rather than membrane-bound.

Despite the fact that we observed higher cf-nDNA levels in samples centrifuged at lower speeds (Fig. 5b), previous studies have shown that lower centrifugation forces do not significantly affect cf-nDNA levels [21, 48]. This discrepancy may be due to residual cell contamination in the low-speed centrifuged plasma samples, which may result in release of DNA during PCR denaturation. In general, the presence of such contamination can artificially inflate cf-nDNA measurements and, thus, potentially bias liquid biopsy results. Therefore, it is highly recommended to adhere to preanalytical instructions in research as well as clinical routine.

Here we assess chronic stress levels in healthy subjects using the standardized and well-established TICS-9 questionnaire to evaluate perceived stress and its effects on cell-free nucleic acids. We have shown that ccf-mtDNA levels are negatively correlated with higher levels of chronic stress (Fig. 6a). In contrast, we did not observe such an effect on cf-nDNA concentrations in conjunction with the perceived stress levels (Fig. 6b). To our knowledge, this is the third study to assess ccf-mtDNA levels in response to stress in vivo, with results conflicting with previous studies that showed elevated ccf-mtDNA levels following stress induction [18]. Trumpff et. al [17] demonstrated an increase in serum ccf-mtDNA concentrations 30 min after induced psychological stress in healthy volunteers, with consistent results in a follow-up examination one month later. However, serum samples may not be suitable to accurately reflect ccf-mtDNA levels, as most of the released mtDNA could be related to the initial clotting process of the platelets. Furthermore, the primer set used by Hummel et al. targets NUMTs as a byproduct, which could potentially contribute to the elevated ccf-mtDNA measurements observed after stress induction.

It is important to note that our study evaluated chronic stress, rather than acute stress, which could lead to different results. Interestingly, a study using a cell culture model of chronic stress simulated by continuous glucocorticoid exposure showed increased ccf-mtDNA levels [49]. Nonetheless, these findings are not necessarily transferable to in vivo conditions, as additional factors such as physiological regulatory mechanisms and inter- or intra variability in stress and immune responses may influence the results.

Although we hypothesize that ccf-mtDNA concentrations might be elevated as a result of mitochondrial dysfunction and damage, the observed negative correlation might suggest complex biological regulatory mechanisms in response to chronic stress. This could include protective adaptions of mitochondria to maintain their functionality and reduce damage to mtDNA. Acute stress typically triggers the “fight or flight” response, which involves the activation of the HPA axis and sympathetic nervous system. This activation can lead to an immune-enhancing effect, characterized by increased proinflammatory cytokine production and increased immune surveillance, whereas chronic stress and prolonged activation of HPA axis can have the opposite effect, leading to immune suppression and dysregulation [50]. Our results indicate that the observed effects are specifically related to mitochondria and mtDNA as we did not find any correlation between the chronic stress levels and cf-nDNA. Further research is needed to investigate the interplay between mitochondrial adaptions, immune regulation and activation and the body’s response to prolonged stress exposure.

## Limitations

Due to the small sample size, it is possible that potential changes in ccf-mtDNA or cf-nDNA were not detected. In this study, ccf-mtDNA levels are close to the LOQ of the direct qPCR assay, indicating that the assay is not yet suitable for determining ccf-mtDNA levels in healthy conditions. Improving the sensitivity of the assay could help quantifying ccf-mtDNA in conditions where concentrations are lower. However, the method could be used to reliably quantify ccf-mtDNA in human blood plasma in other pathological settings.

## Conclusions

In conclusion, we have established a direct and reliable assay for ccf-mtDNA quantification in human blood plasma samples that can be used in liquid biopsy, reducing time and cost of purification. Further research should focus on optimizing this assay for broader clinical use and investigating its applicability in other diseases where liquid biopsy is a potential tool for diagnosis and prognosis. This could be an opening for studying intra- and inter-individual variability of ccf-mtDNA under different conditions and in different pathologies. The reduction in ccf-mtDNA concentrations in relation to perceived chronic stress levels in our healthy cohort suggests regulatory mechanisms specifically related to mtDNA and mitochondrial function, as we did not observe such a correlation in cf-nDNA. Future studies should investigate other biomarkers for stress related manners or in stress related diseases.

## Supplementary Information

Below is the link to the electronic supplementary material.

Supplementary file1 (XLSX 38 KB)

## Data Availability

All data generated and analyzed during this study is included in the electronic supplementary file.

## Abbreviations

BMI: Body mass index
ccf-mtDNA: Circulating cell-free mitochondrial DNA
cf-nDNA: Circulating cell-free nuclear DNA
CLSI: Clinical & Laboratory Standards Institute
Cq: Quantification cycle
CV: Coefficient of variation
dPCR: Digital polymerase chain reaction
DSM-IV: Diagnostic and Statistical Manual of Mental Disorders Fourth Edition
EDTA: Ethylenediaminetetraacetic acid
FDA: Food and Drug Administration
HPA: Hypothalamic–pituitary–adrenal
ISO: International Organization for Standardization
K3: Tripotassium
LINE 1: Long interspersed nuclear element 1
LOB: Limit of blank
LOD: Limit of detection
LOQ: Limit of quantification
ND1: NADH-ubiquinone oxidoreductase chain 1
NTC: Non template control
NUMTs: Nuclear-embedded mitochondrial DNA sequences
qPCR: Quantitative polymerase chain reaction
SCID: Structured Clinical Interview
SD: Standard deviation
TICS-9: Trier Inventory for Chronic Stress
